# Diagnostic analysis of wellness tourism certification bias and public health resource mismatch

**DOI:** 10.3389/fpubh.2026.1870963

**Published:** 2026-07-14

**Authors:** Xiaoyan Liu, Jun Zhao

**Affiliations:** 1 School of Business, Jianghan University, Wuhan, China; 2 School of Innovation and Entrepreneurship, Hubei University of Technology, Wuhan, China

**Keywords:** environmental health, explainable machine learning, health-promoting resource configurations, policy recognition, public health policy

## Abstract

**Introduction:**

Cities promote public health through diverse environmental, service, and access conditions. In China, formal recognition systems shape which health-promoting resource configurations become institutionally visible. These systems rely on standardized templates, so they tend to recognize resources that are easy to measure and compare across regions, while overlooking locally embedded ones. This study asks how heterogeneous configurations translate into recognition signals, and where current systems reach their coverage limits.

**Methods:**

Using panel data from 111 prefecture-level cities in the Yangtze River Economic Belt from 2017 to 2024, the analysis builds a recognition benchmark from national and provincial designation records and applies XGBoost with SHAP under strict temporal extrapolation.

**Results:**

Recognition concentrates on a small set of highly legible indicators. Forest coverage alone accounts for 15.6 percent of resource-base attribution. Enablers such as transport, digital infrastructure, and service capacity show stronger recognition attribution mainly when the resource base is already strong. The study identifies eight city-level pathways and interprets them as policy-aligned, low-readiness, or boundary-advantage positions.

**Discussion:**

Within the Yangtze River Economic Belt, low recognition does not necessarily mean low health value. It can also reflect limits in how current templates read locally embedded resources. These findings refer to institutional visibility, not realized service quality, market performance, or population-health outcomes. The framework helps cities distinguish template alignment, pathway immaturity, and institutional invisibility, supporting differentiated health-and-wellness routes rather than a single recognition-oriented path.

## Introduction

The rise of health and wellness tourism reflects a broader public-health shift from disease treatment toward prevention, health promotion, mental wellbeing, active aging, and lifestyle management. Unlike conventional sightseeing-oriented tourism, it organizes place-based environmental and service resources into experiences intended to maintain or enhance physical, mental, and social wellbeing. Forests, waterscapes, terrain, climate, geothermal environments, traditional health practices, dietary cultures, sports and leisure spaces, and access infrastructures together form city-level health-promoting resource configurations. In this study, these wellness-related practices are treated as a spatial and service-mediated form of health promotion (1–3).

Cities do not develop health-promoting resources through a single universal pathway. Some cities rely on forest ecosystems, protected landscapes, and measurable ecological assets. Others build their health-and-wellness potential around waterscapes, geothermal resources, traditional health-service practices, dietary cultures, sports and leisure spaces, or locally embedded service capabilities. These resources differ not only in substantive health-promoting functions, but also in how they are organized, accessed, experienced, documented, and compared. City-level health-promoting resource development should therefore be understood as a configurational problem rather than as a linear accumulation of isolated resource elements.

This issue is increasingly important for public health. Health-promoting environments shape opportunities for restoration, physical activity, dietary practice, preventive care, social interaction, and service access (4–6). Their relevance extends beyond conventional exposure-based accounts of green or blue space. Forests, waterscapes, terrain, and climate provide the environmental conditions. Traditional health services, cultural-spiritual spaces, nutritional resources, and sports and leisure facilities form the service-experience layer. Transportation, digitalization, and policy support determine access and implementation. The public-health question is therefore not only whether such resources exist, but how cities organize them into accessible and credible health-promoting pathways within environmental health and place-based health promotion.

However, recognition systems do not represent these configurations evenly. Existing designation and recognition programs provide valuable public records of how certain health-and-wellness resources become formally recognized, but these records should not be interpreted as comprehensive measures of health-promoting value. Standardized recognition templates tend to privilege resources that are spatially bounded, measurable, auditable, and comparable across cities (7, 8), such as forest coverage, protected-area status, or administratively classified ecological assets. By contrast, locally embedded resources, including therapeutic waterscapes, traditional health-service practices, dietary cultures, embodied wellness knowledge, or experiential service systems, may support meaningful health promotion while remaining difficult to translate into standardized evidence chains.

Consider two cities in the same region. City A possesses geothermal springs, riparian landscapes, traditional health-service practices, and local food-based wellness resources. City B possesses extensive forest cover whose area, coverage rate, ecological classification, and administrative boundaries are easier to document and verify. If City B receives stronger formal recognition, the difference does not necessarily mean that City A lacks health-promoting value. It may instead indicate that current recognition templates more readily read forest-based resources than locally embedded, service-mediated, or experiential advantages. The key issue is therefore not how cities can maximize recognition, but how selective recognition systems reveal different development positions and distinguish resource constraints from institutional under-recognition.

Existing public-health research has examined the health relevance of green space and blue space (5, 9), built environments (6), land use and land cover patterns linked to population health (10, 11), and place-based health promotion (4). However, less attention has been paid to how heterogeneous health-promoting resource configurations, many of which are spatially anchored in city-level land use, are selectively represented within standardized policy-recognition systems. Recognition records are often treated as indicators of development success or policy support, while their role as selective recognition signals remains insufficiently examined. This creates a risk of conflating low recognition with low health-promoting value and of overlooking cities whose locally meaningful resources fall near the institutional coverage boundary. Accordingly, this study uses publicly observable recognition records as policy-recognition signals rather than as outcomes to be optimized.

Three questions guide the analysis. First, how do standardized recognition systems translate city-level health-promoting resource configurations into policy-recognition signals? Second, do enabling conditions show stronger recognition-attribution patterns only when resource bases are already strong and legible? Third, can cities be sorted into diagnostic positions that distinguish template-compatible advantages, low-readiness configurations, and locally meaningful but under-recognized boundary advantages?

The study contributes on three connected fronts. First, it reframes recognition records from taken-for-granted quality labels into policy-recognition signals that reveal how standardized templates translate heterogeneous resource configurations into uneven institutional legibility. Second, it operationalizes the institutional coverage boundary as a measurable diagnostic concept and links it to three strategic city positions, thereby transforming recognition asymmetries from a qualitative observation into city-level traceable indicators. Third, it develops a public-health-oriented diagnostic framework for differentiated development by separating resource constraints from weak institutional legibility, so that low recognition can be interpreted as a diagnostic signal rather than as direct evidence of low value.

## Theoretical framework

Health-promoting resource configurations are formed through the interaction of place-based environmental conditions, service-experience resources, and enabling infrastructures. Their public-health relevance does not depend only on the presence of individual resources, but on whether these resources can be organized into accessible, credible, and institutionally legible pathways. For cities, this means that health-promoting resources operate simultaneously as substantive local assets and as objects of selective institutional representation. A city may possess forests, waterscapes, traditional health practices, dietary resources, sports facilities, or therapeutic environments, but these assets do not enter public recognition systems equally.

The framework combines a resource-configuration perspective with an institutional-filtering perspective. The resource-based view explains why heterogeneous bundles rather than isolated factors shape city-level advantage structures. Institutional theory explains why standardized recognition systems do not read all advantages equally, because they impose selective evidentiary requirements on what can become institutionally legible. Together, these perspectives shift the analysis from what cities possess to how their resource configurations are recognized.

### Resource heterogeneity and conditional complementarity

Natural environments constitute a well-documented class of health-promoting resources. Forest exposure has been associated with reduced stress, improved immune function, and lower all-cause mortality. Blue-space environments support restorative experiences and physical activity. Climate and terrain conditions shape chronic disease risk, thermal comfort, and therapeutic opportunity (5, 9). At the city level, however, the value of these resources depends not only on their presence but on how they are organized into usable pathways and connected to services, infrastructures, and implementation capacities. Recent umbrella and policy-oriented reviews further confirm that diverse natural environments support multiple dimensions of population health (12, 13).

The resource-based view argues that durable advantages arise from heterogeneous resources and capabilities that are valuable, rare, difficult to imitate, and difficult to substitute (14). Later work further emphasizes that value is context-dependent and combinatorial: resources generate returns not simply through possession, but through their joint deployment with complementary assets and organizational capacities (15). This logic is highly relevant to city-level health-and-wellness development, where health-promoting value often emerges from the combination of environmental resources, service systems, cultural practices, accessibility, and governance capacity.

For health-promoting resource configurations, the key implication is that no single resource type should be treated as a complete proxy for city-level potential. Forests, lakes, mountains, hot springs, climate conditions, traditional medicine, nutritional resources, sports and leisure facilities, and cultural-spiritual spaces each support different forms of restoration, activity, prevention, care, and social interaction. Their public-health relevance depends on how they are combined and made accessible. A city with strong natural endowments but weak service capacity may struggle to transform resources into usable health-promoting opportunities. Conversely, a city with relatively strong service capabilities but weak or poorly legible resource bases may remain weakly represented within standardized recognition systems. This leads to a first expectation regarding recognition concentration.

**Proposition 1 (sparse recognition structure)**. Recognition contributions concentrate on a small number of highly legible indicators rather than distributing evenly across resource dimensions.

### Institutional filtering and policy recognition asymmetry

The resource-based perspective explains why cities differ in their health-promoting resource configurations, but it leaves open a further question. Why do some configurations become more visible in recognition systems than others? Institutional theory offers a second explanation. Institutions shape the categories, rules, and evidentiary standards through which practices become legitimate and actionable (16–18). Within recognition systems, these standards operate through evaluation templates, designation criteria, documentation requirements, and review procedures. They determine which resource attributes can be turned into auditable evidence and which remain weakly legible within standardized administrative routines. Certification research points in a similar direction, showing that standards can shape organizational behavior and legitimacy-seeking beyond their formal administrative moment (7).

Recognition systems are, in this sense, selective policy instruments rather than neutral evaluators of underlying quality. They do not simply discover value that is already there. Instead, they define which forms of value can be formally recognized. Attributes that are spatially bounded, measurable, administratively documented, and comparable across cities are more likely to enter the recognition field. Forest coverage, ecological classification, protected-area status, and formal designation records fit this evidentiary template. Locally embedded traditional health practices, embodied service knowledge, dietary cultures, therapeutic waterscapes, and service-mediated experiences may be important for health promotion, yet they are far harder to standardize, audit, and compare.

Part of this selectivity reflects the practical demands of administrative comparability and policy accountability. It also produces recognition asymmetry. Some cities are recognized more strongly partly because their resources are easier to document and verify, while others may hold distinctive health-promoting assets that remain weakly represented because their dominant resource attributes fall outside what current templates can effectively capture.

The institutional coverage boundary captures this mismatch. It marks the point at which locally meaningful health-promoting resources fail to register as strong policy-recognition signals under standardized templates. The boundary is not a claim that such resources lack value. It identifies where recognition templates remain insufficiently sensitive to heterogeneous local assets.

### The R-S-E strategic configuration framework

Building on the above arguments, this study organizes city-level health-promoting resource configurations into an R-S-E framework: natural endowment resources, service-experience resources, and enablers. The framework distinguishes resource types by their functional roles in the formation and institutional representation of health-promoting development pathways.

Natural endowment resources (R) refer to place-based environmental conditions that provide the ecological and spatial foundation for health promotion. They include forests, waterscapes, terrain, climate, geothermal resources, environmental quality, and related natural conditions. These resources are often spatially sticky and difficult to reproduce. Some of them, especially forest-related indicators, are highly compatible with standardized evidence templates because they can be measured through area, coverage rate, ecological classification, and administrative boundaries. These natural conditions are also widely treated as health-promoting environmental assets in public-health research (5, 19–21). They also overlap with land use and urban land-cover concerns, since the distribution of forests, waterscapes, green infrastructure, and other environmental assets helps structure the spatial availability of restorative environments, opportunities for physical activity, and population exposure to environmental risks (11, 22). While their health relevance is well established, what remains underexamined is how recognition systems differentially represent these environmental health assets.

Service-experience resources (S) refer to resources that translate environmental and cultural conditions into usable health-promoting experiences. They include traditional health services, traditional medicine heritage, sports and leisure activities, cultural-spiritual spaces, dietary and nutritional resources, and related service capacities. Compared with natural endowments, these resources are often more locally embedded, experiential, and organizationally mediated. Their health-promoting value may be substantial, but their institutional representation depends on whether they can be converted into auditable and comparable evidence.

Enablers (E) refer to access and implementation conditions that support the transformation of resources into recognized development pathways. They include transportation infrastructure, digitalization, economic foundations, service capacity, policy supply, and other implementation conditions. Enablers do not necessarily generate recognition independently. Their attribution patterns are likely to depend on the strength and legibility of the underlying resource base. This framework leads to a second expectation. If enablers work primarily through the resource base rather than independently from it, their contribution patterns should be conditional rather than universal.

**Proposition 2 (conditional amplification)**. Enabler attribution patterns depend on the resource base. Where R or S resources are strong and legible, enablers show stronger positive recognition attribution. Where the resource base is weak, enabler attribution remains limited or shifts toward compensatory patterns.

Finally, the R-S-E framework implies that cities may follow multiple development pathways. Because resource bundles differ across cities, policy recognition should not be expected to arise from a single universal configuration. However, these pathways are not equally legible within current recognition systems, suggesting differentiated diagnostic positions.

**Proposition 3 (multi-pathway diagnosis)**. City-level resource configurations form multiple pathways that occupy different positions relative to the dominant recognition template. Three diagnostic positions emerge: policy-aligned, low-readiness, and boundary-advantage.

**Figure 1:**
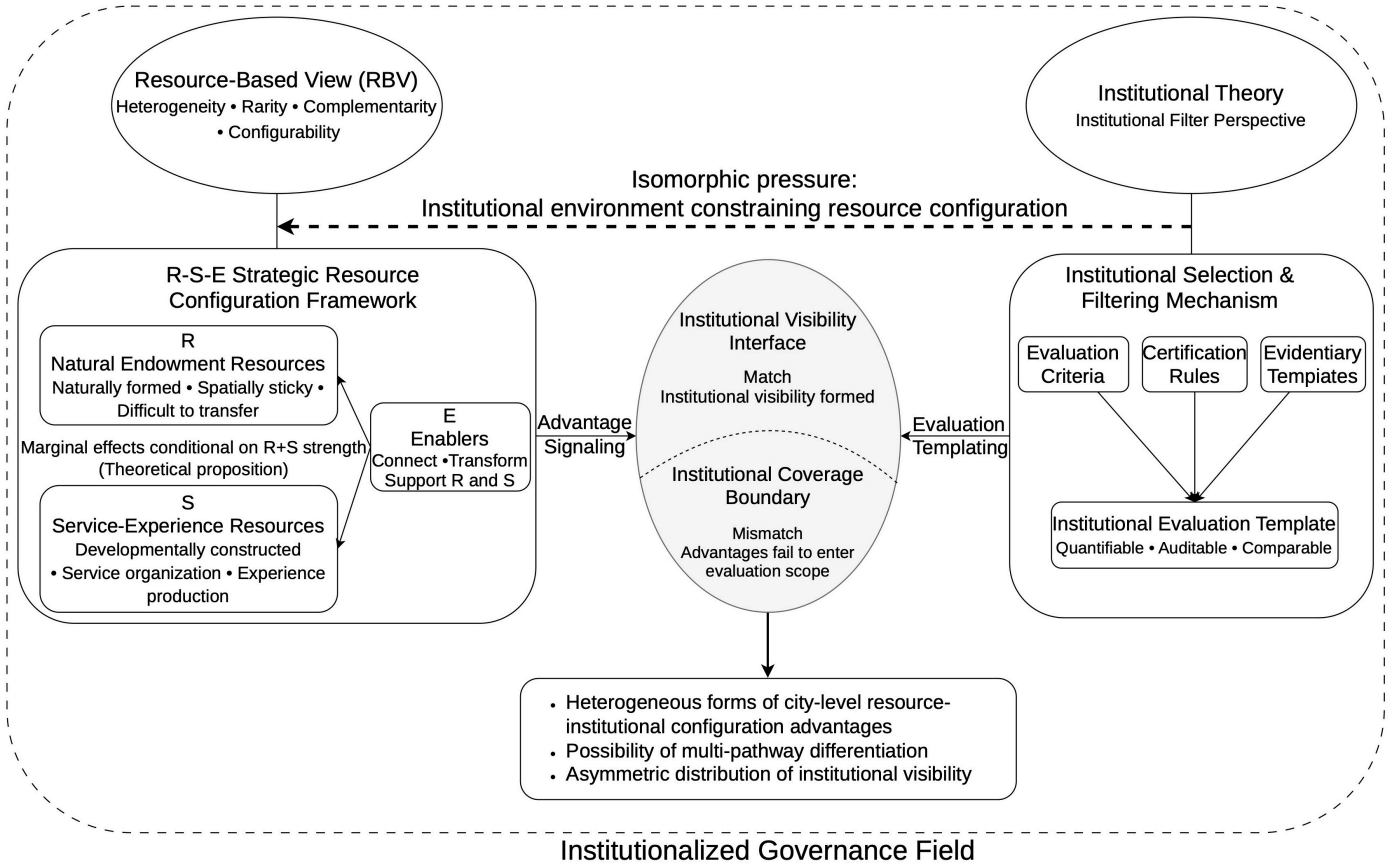
Theoretical framework.

Figure 1 summarizes the theoretical framework. The left side represents heterogeneous R-S-E resource configurations. The middle layer represents policy-recognition processes, where standardized templates translate selected resources into auditable signals. The right side represents diagnostic positions. Cities whose resource configurations fit dominant templates appear policy-aligned. Cities with recognizable but insufficiently mature resource configurations are interpreted as low-readiness cases. Cities with distinctive but weakly represented resources are positioned near the institutional coverage boundary and interpreted as boundary-advantage cases.

## Research design and methods

### Study area and data sources

This study examines 111 cities in the Yangtze River Economic Belt, a region spanning eastern, central, and western China across 11 provincial administrative regions. The sample comprises prefecture-level cities together with two provincial-level municipalities, which are treated as city-level observation units for cross-city comparability. The Belt provides a suitable empirical setting because it contains substantial variation in forests, waterscapes, terrain, climate, traditional health resources, medical and service capacities, transportation conditions, and policy supply. During the study period, multiple national and provincial wellness-related designation programs generated observable records across cities and years. These characteristics make the region appropriate for examining how heterogeneous city-level health-promoting resource configurations are represented within standardized recognition systems. This heterogeneity is important for public-health-oriented wellness research because it allows the study to examine how different cities organize environmental, service-experience, and enabling conditions into differentiated health-promoting pathways.

Research data derive from three categories. The first category includes socioeconomic and development data, drawn primarily from the China City Statistical Yearbook and provincial or municipal statistical bulletins. The second category includes medical, ecological, environmental, and climate-related data, drawn from health statistics yearbooks, environmental bulletins, and scientific meteorological products. The third category includes policy-recognition records, compiled through systematic searches of national and provincial government websites related to wellness-related designation programs, including forest wellness bases, traditional Chinese medicine health tourism sites, and integrated wellness demonstration programs.

The sample period spans 2017 to 2024, beginning with the launch of national-level health tourism demonstration site initiatives and the announcement of the first batch of designated sites in 2017 and extending through subsequent rounds of recognition at both national and provincial levels. Lists collected through the end of 2024 cover both general programs (e.g., health and wellness tourism demonstration sites) and specialized programs (e.g., forest health bases, traditional Chinese medicine health tourism demonstration sites), recording designation year, administrative level (national or provincial), and spatial coverage (whole-territory or site-specific). Policy supply intensity was measured from annual municipal government work reports. The corpus was first retrieved from the Government Work Report collection of the People's Database (Renmin Database). Reports that were missing from the database, incomplete, or required source verification were manually supplemented from publicly released prefecture-level municipal government reports, including official government websites and government-information-disclosure portals. Appendix A1 reports the dictionary, matching rules, normalization procedure, and validation check. Multi-source data are matched on city codes and years, with inconsistent indicator calibrations standardized uniformly; a small number of missing values are linearly interpolated, while structurally missing variables are handled through sample exclusion or indicator substitution rather than interpolation.

To evaluate generalization ability, the data are partitioned into three temporal segments: a training period (2017–2021, 5 years, 555 observations), a validation period (2022, 1 year, 111 observations), and a test period (2023–2024, 2 years, 222 observations), ensuring that training relies entirely on historical patterns while evaluation is conducted on out-of-sample future periods. All preprocessing parameters, including standardization parameters for explanatory variables and Min-Max scaling parameters for the external criterion, are fitted solely on training-period data and applied forward to the validation and test periods, preventing information leakage at source.

### Variables and measurement

#### Recognition benchmark construction

Comparable objective data on city-level wellness-tourism development are not available through a unified public city-year system in China. Variables such as wellness-tourism visitation, spending, length of stay, service quality, repeat participation, and health improvement are held by destinations, platforms, or service providers, and they are not comparable across cities or years. Accordingly, the dependent variable is not a measure of actual wellness-tourism development.

The dependent variable is a policy-recognition benchmark constructed from publicly released national and provincial designation records. The benchmark represents cumulative policy-recognition intensity and measures how city-level health-promoting resource configurations become institutionally visible within existing recognition systems. Official recognition records provide a traceable and cross-city comparable account of how standardized institutions classify, prioritize, and support different health-promoting resources. Its analytical value lies in revealing which city-level resource configurations are formally registered under standardized evaluation templates, rather than in capturing population-health outcomes, service quality, market performance, or comprehensive resource value. In this sense, recognition records are treated as policy-recognition signals rather than quality labels or direct measures of health value. A higher benchmark value therefore indicates stronger institutional visibility within the observed recognition system, not stronger substantive development or better public-health performance.

This benchmark avoids the circular reasoning of internal weighting methods such as AHP or entropy weighting, while aligning SHAP decomposition with its theoretical purpose: to show how observable institutional preferences shape city-level contribution structures. It therefore reconstructs how the policy-recognition system represents heterogeneous resources, rather than estimating their true health value or establishing causal links to realized health outcomes. Designation records are treated as the object of analysis, not as proxies for that true health value.

To ensure replicability and minimize subjectivity, the benchmark scoring relies on publicly observable attributes of designation records, including administrative level, spatial coverage type, and designation category. A base score is first assigned according to the highest-level general designation a city holds each year. Holding at least one national-level general designation yields a score of 5, holding only provincial-level general designations yields 3, and holding neither yields 0. These weights are used only to construct a consistent policy-recognition system, and they do not imply proportional differences in substantive health-promoting value across cities. Cumulative bonuses for both general and specialized (thematic) designations are then added as follows (Equation 1).


$$\begin{array}{l} \begin{array}{c} \text{B}\text{o}\text{n}\text{u}{\text{s}}_{\text{i},\text{t}}=\underset{l}{\sum }\underset{s}{\sum }\left(n_{\text{g}\text{e}\text{n},i,t}^{l,s}\cdot w_{\text{g}\text{e}\text{n}}^{l}\cdot \beta^{s}+\overset{J}{\underset{j=1}{\sum }}n_{j,i,t}^{l,s}\cdot w_{\text{s}\text{u}\text{b}}^{l}\cdot \beta^{s}\right) \end{array} \end{array}$$ (1)

where $n_{\text{g}\text{e}\text{n},\text{i},\text{t}}^{l,s}$ is the cumulative count of general designations; $n_{j,i,t}^{l,s}$ is the cumulative count of the *j*-th specialized designation; *l*∈{*nat, prov*}; and *s*∈{*full, point*}. Weights $w_{\text{g}\text{e}\text{n}}^{l}$, $w_{\text{s}\text{u}\text{b}}^{l}$, and spatial coefficient β^*s*^are assigned as follows: $w_{\text{g}\text{e}\text{n}}^{\text{n}\text{a}\text{t}}=3$, $w_{\text{g}\text{e}\text{n}}^{\text{p}\text{r}\text{o}\text{v}}=1.5$, $w_{\text{s}\text{u}\text{b}}^{nat}=2.0$, $w_{\text{s}\text{u}\text{b}}^{\text{p}\text{r}\text{o}\text{v}}=1.0$; β^full^ = 1.0, β^point^ = 0.8. Specialized designations across different themes *j* share the same weight structure.

These weights serve as ordinal proxies for institutional visibility rather than as precise fiscal or health-value multipliers. National recognition receives a higher weight than provincial recognition because it carries higher-level administrative endorsement, broader cross-regional comparability, and closer coupling with national policy priorities. General designations receive a base score because they recognize broader city-level and destination-level health-and-wellness pathways, while specialized designations are treated as cumulative additions because they reveal institutional attention to more specific resource types. Whole-territory recognition receives a higher spatial coefficient than site-specific recognition because it signals broader administrative coverage. These differences capture ordered variation in policy-recognition intensity, and they do not imply proportional differences in health-promoting value, service quality, or population-health benefit.

Reward differentials between national and provincial designations vary across regions, which precludes a universally precise multiplier. Even so, the subsidy policies of many cities indicate a national-to-provincial spacing of roughly 2 to 1, as reflected in the reward standards for national and provincial demonstration programs in Wuhan and Sanming. Sensitivity tests using a count-based specification and forest-theme downweighting show that the main feature-contribution structures remain broadly stable across alternative benchmark specifications. The composite external criterion is then defined as follows (Equation 2):


$$\begin{array}{l} \begin{array}{c} Y_{o,i,t}=\text{B}\text{a}\text{s}{\text{e}}_{\text{i},\text{t}}+\text{B}\text{o}\text{n}\text{u}{\text{s}}_{\text{i},\text{t}}+\epsilon ,\;\epsilon =0.1 \end{array} \end{array}$$ (2)

where ϵ is a smoothing constant that prevents undefined logarithmic or power transformations at zero values.

#### Explanatory variables

Drawing on the R–S–E strategic resource configuration framework developed above, supply-side conditions for city *i* in year *t* are operationalized as an explanatory variable vector *X*_*i, t*_ comprising 58 city-year indicators organized into three categories: natural endowment resources (R, 6 subdimensions, 21 indicators), service-experience resources (S, 5 subdimensions, 18 indicators), and enabler resources (E, 5 subdimensions, 19 indicators). Definitions, subdimension assignments, and unit calibrations for each indicator are provided in Appendix A2.

Explanatory variables undergo unified preprocessing before entering models: indicators where higher values represent worse conditions are directionally reversed; continuous variables with significant right skewness are log(*x*+1) - transformed to mitigate extreme-value influence; continuous variables are then Z-score standardized. Binary dummy variables remain untransformed.

### Modeling and analysis strategy

The analysis strategy comprises two stages. The modeling stage learns the non-linear mapping between multidimensional city-level health-promoting resource configurations and the policy-recognition signal. The interpretation stage decomposes this learned mapping into city-level feature contribution structures for proposition testing, dominant-driver identification, pathway typology, and coverage-boundary diagnosis.

#### Stock regression and flow (Event) regression

To identify mechanisms of institutional recognition from complementary perspectives, two models are constructed. The stock model characterizes structural correspondence in the cumulative dimension, identifying which resource-configuration combinations correspond to stronger cumulative representation within the policy-recognition system (Equation 3):


$$\begin{array}{l} \begin{array}{c} Y_{o,i,t}^{\text{n}\text{o}\text{r}\text{m}}=f_{\text{S}\text{t}\text{o}\text{c}\text{k}}\left(X_{i,t}\right)+\varepsilon_{i,t} \end{array} \end{array}$$ (3)

Cumulative indicators, however, exhibit strong serial persistence, so associations captured by the stock model may partly reflect status inertia rather than marginal mechanisms. The flow model strips away part of this inertia by focusing on marginal conditions associated with whether a new entry into the institutional reference field occurs. Because the vast majority of cities receive no new designation in any given year, incremental data display pronounced zero-inflation, and directly modeling continuous increments causes predictions to degenerate toward trivial near-zero values; the increment is therefore transformed into a binary event signal. Let $E_{i,t}=1\left\{\Delta Y_{o,i,t}^{\text{n}\text{o}\text{r}\text{m}}>0\right\}$ indicate whether city *i* experiences new recognition in year *t*, where $\Delta Y_{o,i,t}^{\text{n}\text{o}\text{r}\text{m}}=Y_{o,i,t}^{\text{n}\text{o}\text{r}\text{m}}-Y_{o,i,t-1}^{\text{n}\text{o}\text{r}\text{m}}$. The flow model is specified as follows (Equation 4):


$$\begin{array}{l} \begin{array}{c} \text{P}r\left(E_{i,t}=1|{\text{X}}_{i,t}\right)\approx f_{\text{F}\text{l}\text{o}\text{w}}\left({\text{X}}_{i,t}\right) \end{array} \end{array}$$ (4)

Because the flow model requires information from the previous period, its effective training period begins in 2018. Together, the two models provide complementary evidence on persistent representation and dynamic entry into the institutional reference field. The stock model captures cumulative representation, while the flow model captures marginal breakthroughs in the reference system.

#### Learner selection and training strategy

XGBoost, a gradient boosting tree algorithm, is selected as the primary learner for three reasons (23): its regularized objective function explicitly penalizes tree complexity and leaf node weights, suiting the small-sample high-dimensional setting of this study (*N* = 888, *p* = 58) and suppressing overfitting risks in temporal extrapolation. It captures nonlinear contribution patterns and variable interactions without presupposing functional forms, accommodating potential thresholds and conditional contribution patterns between resource bases and enabling support, and it is natively compatible with TreeSHAP, facilitating subsequent feature contribution decomposition.

The stock model employs a regression objective function and the flow model a binary classification objective function. Four hyperparameters were tuned via year-based grouped five-fold cross-validation within the training period (2017–2021), using grid search over maximum tree depth, learning rate, subsample ratio, and regularization coefficients. Model selection was based on cross-validation performance within the training period and checked against the independent validation year (2022). After the optimal hyperparameters were determined, the final model was refitted on the merged training-and-validation data (2017–2022), while the test period (2023–2024) remained fully held out for temporal extrapolation.

To quantify the information gain provided by feature variables and assess the necessity of nonlinear modeling, two formal baseline models are established alongside an additional reference anchor. The linear baseline uses Lasso regression for the stock task and logistic regression for the flow task (both with *L*_1_ regularization): if XGBoost significantly outperforms these linear baselines in the test period, non-linear patterns that linear models cannot adequately capture are indicated. The constant-probability baseline calibrates the predictive floor under no feature information for the flow task by assigning all observations a predicted probability equal to the event rate in the merged training-and-validation sample, ${\overset{-}{E}}_{train+valid}$. Because the stock-task criterion is cumulative and exhibits strong serial persistence, the results additionally report a persistence reference ${Ŷ}_{i,t}^{pers}=Y_{o,i,t-1}^{norm}$ to calibrate the predictive ceiling attributable to status continuation; this reference is not treated as a formal baseline because it relies on lagged *Y* rather than feature variables *X* and therefore falls outside the *X*_*i, t*_→*Y* mechanism-attribution objective. Model performance is evaluated primarily by test-period R^2^ and MAE, with RMSE reported as a supplement. These metrics are used to ensure that the learned mapping is sufficiently stable for diagnostic decomposition rather than to optimize recognition outcomes. High predictive accuracy is therefore a credibility condition for SHAP-based interpretation rather than the substantive objective of this study.

### Interpretable decomposition and urban diagnosis (SHAP)

The SHAP (SHapley Additive exPlanations) method is employed to decompose model outputs into additive feature-level contributions (24). For any city-year observation (*i, t*), the stock model output is decomposed as follows (Equation 5):


$$\begin{array}{l} \begin{array}{c} {Ŷ}_{\left(i,t\right)}=\varphi_{0}+\Sigma_{\left(k=1\right)}^{p}\varphi_{\left(i,t,k\right)} \end{array} \end{array}$$ (5)

where φ_*i, t, k*_ is the marginal contribution of the *k*-th feature to the predicted value and φ_0_ is the baseline expectation. For the flow model the interpretation target is the log-odds of the new-event probability (Equation 6):


$$\begin{array}{l} \begin{array}{c} logit\left(p{ˆ}_{\left(i,t\right)}\right)=\varphi_{0}^{\left(E\right)}+\Sigma_{\left(k=1\right)}^{p}\varphi_{\left(i,t,k\right)}^{\left(E\right)} \end{array} \end{array}$$ (6)

Because XGBoost classification models output log-odds by default, TreeSHAP performs decomposition directly on the margin scale. The unit of $\varphi_{i,t,k}^{\left(E\right)}$ is thus a log-odds contribution, preserving additivity across the entire value domain. To characterize the overall preference structure of the policy-recognition system, the global importance of a feature is defined as its mean absolute contribution during the test period (Equation 7):


$$\begin{array}{l} \begin{array}{c} I_{k}=\left(1/|T_{t}est|\right)\Sigma_{\left(i,t\in T_{t}est\right)}|\varphi_{\left(i,t,k\right)}| \end{array} \end{array}$$ (7)

*I*_*k*_ reflects the average contribution intensity of a feature within the institutional reference-signal mapping; for subdimensional analysis, *I*_*k*_ values within a subdimension are summed to provide a measure of global legibility. *I*_*k*_ represents attribution intensity within the model's logic and is distinct from causal effects or XGBoost's built-in Gain/Split importance. For each observation the contribution vector **φ**_**i**, **t**_ serves as the primary input for pathway identification and diagnostic analysis. SHAP contributions are interpreted as contributions to the learned reference-signal mapping rather than as causal effects on health outcomes, policy success, or substantive resource value. Positive SHAP values indicate that a resource attribute is favorably represented within the current policy-recognition system; negative values indicate that the attribute is weakly represented or constraining within that mapping?

### Proposition testing and diagnostic strategy

The three propositions are evaluated through model-attribution diagnostics that characterize how the learned policy-recognition mapping concentrates, amplifies, and differentiates resource contributions, rather than through causal estimation of effects on recognition or health outcomes.

**Proposition 1: sparse policy-recognition structure**. To determine whether positive recognition contributions concentrate within a few highly legible resource attributes, a Dominance Index is defined on the resource-base set *R*∪*S* to measure the concentration of positive contributions (Equation 8):


$$\begin{array}{l} \begin{array}{c} Dom_{\left(i,t\right)}=max_{\left(k\in \left(R\cup S\right)\right)}\varphi_{\left(i,t,k\right)}^{+}/sum_{\left(k\in \left(R\cup S\right)\right)}\varphi_{\left(i,t,k\right)}^{+} \end{array} \end{array}$$ (8)

where $\varphi_{\left(i,t,k\right)}^{+}=\max\left(\varphi_{\left(i,t,k\right)},0\right)$ is the positive contribution component. An index value approaching 1 indicates that positive contributions are dominated by one or very few resource elements (sparse driver structure), while a value approaching 1/|*R*∪*S*| indicates contributions distributed evenly across multiple resources (balanced driver structure). Classification thresholds are determined from the empirical distribution of *Dom*_*i, t*_.

**Proposition 2: conditional amplification of enablers**. To test whether enablers (E) exhibit conditional attribution patterns dependent on the resource base, grouped tests are conducted simultaneously on both stock and flow models. Observations are divided into high and low groups based on the median of the summed standardized values of the 39 variables. Both models share this grouping logic but differ in temporal alignment. The stock perspective uses period-*t* grouping to capture cross-sectional structural differences, whereas the flow perspective uses period-(*t*−1) variables to ensure that grouping information temporally precedes the current event, thereby mitigating endogeneity risks. When both scales show the same direction (amplification or compensation), the conditional legibility pattern is robust. When directions diverge, the pattern depends on scale.

**Proposition 3: multi-pathway diagnosis and coverage-boundary differentiation**. To diagnose heterogeneous pathways of health-promoting resource configurations within the policy-recognition system, pathway typology uses the contribution structures of test-period city-year observations as input.? SHAP contributions from the resource base categories are first aggregated by subdimension, retaining only positive contributions to characterize each city's resource advantage profile (hereafter, “advantage bundles”), and then Z-score standardized to eliminate scale-dependent biases in distance metrics.

Institutional filtering often causes a few highly legible dimensions to dominate the contribution structure, so full-sample clustering tends to be skewed by these dominant distance structures, compressing the fine-grained differentiation among low-visibility advantage combinations. A three-step strategy is therefore adopted: dominance diagnosis, conditional stratification, and within-stratum clustering. The global legibility of each subdimension (summed test-period *I*_*k*_) and its observation-level dominance frequency (defined as the subdimension with the highest positive contribution share in a single observation) are first calculated; if a subdimension d^*^ ranks high on both metrics, the sample is stratified into two layers at the median of its positive SHAP contributions, and Ward hierarchical clustering is implemented within each stratum, searching for the optimal solution within a preset range (*K* = 3 − 8). If no such dominant subdimension is identified, Ward clustering is applied directly to the full sample. The final solution is determined by integrating silhouette coefficients, minimum cluster size constraints, and cross-year stability (the consistency rate of pathway assignment for the same city between 2023 and 2024).

Building on this typology, observations potentially falling near the institutional coverage boundary are further identified. Subdimensions are ranked by global visibility, with the bottom 50% constituting the low-legibility set. An observation is classified as a coverage-boundary case if its dominant subdimension falls into this set. This diagnostic criterion captures the mismatch between local significance and weak global legibility within standardized recognition systems. Pathways are then interpreted through three strategic positions. Policy-aligned pathways are those whose dominant dimensions fit the high-legibility recognition template and show relatively strong recognition outcomes. Low-readiness pathways are those whose dominant dimensions are not necessarily illegible but whose overall recognition levels remain low, indicating insufficient resource strength or pathway maturity. Boundary-advantage pathways are those whose dominant dimensions fall into low-legibility sets despite meaningful local resource advantages. In robustness checks, the low-legibility set is held fixed to the BASE specification to ensure comparability across criterion variants.

## Empirical results and diagnostic analysis

### Descriptive statistics

**Figure 2:**
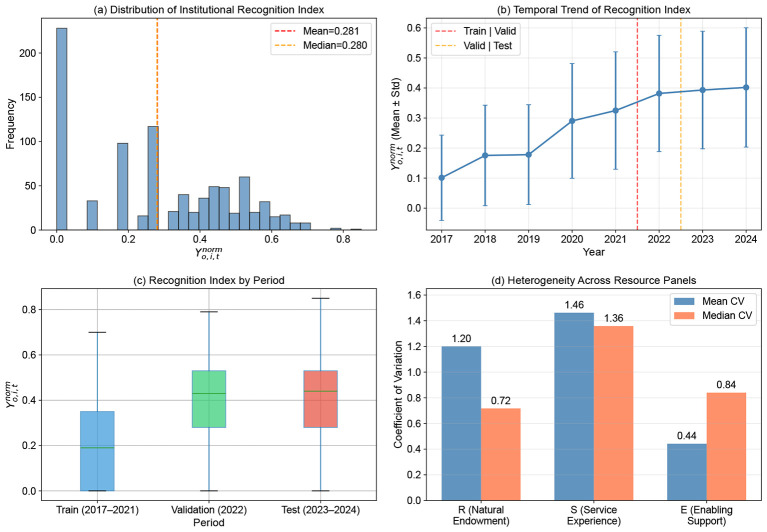
Descriptive statistics. **(a)** Distribution of the normalized institutional reference index. **(b)** Yearly mean of the normalized institutional reference index with error bars indicating ±1 standard deviation across cities; vertical dashed lines mark Train/Validation/Test boundaries. **(c)** Box plots by period: Train (2017–2021), Validation (2022), Test (2023–2024). **(d)** Coefficient of variation by panels R/S/E; bars report mean CV and labeled values report median CV.

Figure 2 presents the basic characteristics of the institutional reference benchmark and explanatory variables in terms of distributional form, temporal evolution, period comparison, and panel heterogeneity.

(a) The institutional reference benchmark exhibits a zero-inflated, right-skewed distribution. Most observations cluster near zero, and only a small number extend into the right tail. This pattern indicates highly uneven cumulative representation.

(b) Annual means rise in a stepwise fashion, with a notable increase around 2020 followed by slower growth. Cross-city dispersion does not converge significantly as means rise, so overall elevation with persistent differentiation characterizes the entire sample period. Vertical lines mark the training/validation/test boundaries.

(c) Period box plots confirm this trend: medians and upper quartiles shift upward in the validation and test periods relative to the training period, yet lower tails remain close to zero, indicating that low-representation observations persist in later periods.

(d) Coefficients of variation across the three panels show that cross-city heterogeneity stems primarily from the resource base rather than enablers. S (service-experience) has the highest mean CV at 1.36, followed by R (natural endowment) at 1.20 and E (enabler) at 0.44. Subdimensional CVs are detailed in Appendix A2.

### Model performance and out-of-sample credibility

Diagnostic decomposition and pathway typology based on SHAP contribution vectors require that the mapping between city-level resource configurations and the policy-recognition system remains stable out of sample. Table 1 summarizes performance in the strict temporal extrapolation test period (2023–2024, *N* = 222).

**Table 1:** Strict out-of-time performance for the institutional reference-signal mapping in the test period (2023–2024).

Model	R^2^	MAE	RMSE	AUC	PR-AUC	LogLoss / Brier	*N*
Panel A. Stock (regression): predicting $Y_{\text{o},\text{i},\text{t}}^{\text{n}\text{o}\text{r}\text{m}}$							
Persistence reference	0.981	0.021	0.027				222
XGBoost (Final)	0.756	0.082	0.097	—	—	—	222
Lasso (Linear baseline)	0.394	0.135	0.153	—	—	—	222
Panel B. Flow (event classification): predicting $E_{i,t}=1\left\{\Delta Y_{\text{o},\text{i},\text{t}}^{\text{n}\text{o}\text{r}\text{m}}>0\right\}$							
XGBoost (Final)	—	—	—	0.746	0.503	0.517 / 0.171	222
Logistic (L1 baseline)	—	—	—	0.694	0.395	0.529 / 0.176	222
Constant-probability baseline ($\hat{p}=0.429$)	—	—	—	0.500	0.221	0.623 / 0.215	222

The Persistence reference in Panel A uses lagged recognition status as a calibration anchor for serial inertia. It is not a formal feature-based baseline because it relies on lagged recognition information rather than the explanatory variables. Baselines in Panel B reflect the combined training-validation event rate and the test-period prevalence (0.221), respectively. LogLoss and Brier scores assess probability calibration under base-rate shifts.

The stock task characterizes structural correspondence between the cumulative policy-recognition system and multidimensional city-level resource configurations. Because the criterion is cumulative, strong serial persistence exists between adjacent years: simply using the previous-period value as the current prediction ($\hat{Y_{i,t}}=Y_{o,i,t-1}^{norm}$) yields a test-period *R*^2^ of 0.981, calibrating the variance ceiling explainable by state continuation alone. Against this backdrop, XGBoost based on current-period feature variables achieves R^2^ = 0.756, significantly outperforming the linear Lasso baseline, indicating that non-linear and interactive patterns exist in the mapping between city-level resource configurations and the policy-recognition system. This provides a credible foundation for subsequent SHAP-based diagnostic decomposition.

The flow event task focuses on marginal entry patterns, namely whether a new record enters the institutional reference field in a given year. Because the test-period event rate (0.221) is substantially lower than the training-period rate (0.429), PR-AUC serves as the primary criterion: XGBoost achieves PR-AUC = 0.503 (AUC = 0.746), exceeding the logistic (*L*_1_) baseline (PR-AUC = 0.395) and the no-skill baseline (≈0.221), confirming stable out-of-sample information gain for discriminating new entries into the reference field. The flow model is positioned as an auxiliary perspective to the stock model in subsequent analysis, used for cross-scale validation in Proposition 2 conditional testing. These metrics establish the stability required for SHAP-based diagnostic decomposition.

### Global feature importance and visibility structure

**Figure 3:**
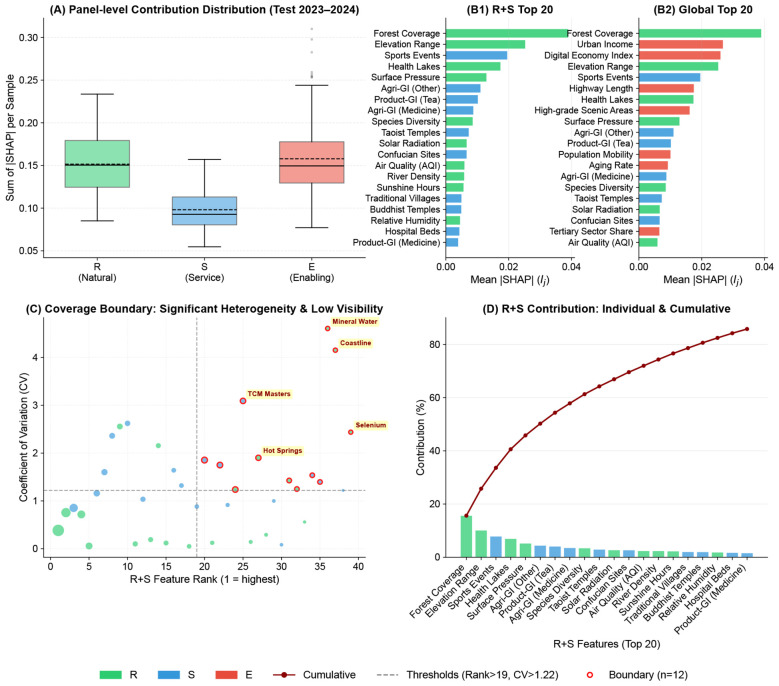
Global reference-signal visibility structure revealed by SHAP. **(a)** Panel-level distribution of summed absolute SHAP contributions for R, S, and E panels in the test period (2023–2024); dashed lines indicate panel means. **(b1)** Top 20 resource-base features from R+S ranked by mean absolute SHAP contribution. **(b2)** Global top 20 features ranked by mean absolute SHAP contribution after incorporating enabler variables. **(c)** Coverage-boundary candidate identification based on high cross-city heterogeneity and low global visibility; dashed lines indicate screening thresholds and red outlined points mark boundary candidates. **(d)** Individual and cumulative contribution shares of the top 20 R+S features, showing that the first five features contribute nearly half and that contributions are concentrated in a small number of highly legible resource indicators.

Figure 3 synthesizes the reference-signal contribution structure based on SHAP decomposition of the stock model in the test period across four dimensions: panel contribution distribution, feature importance ranking, coverage-boundary candidate identification, and contribution concentration.?

**Panel contribution distribution (Panel A)**. The resource base panels (R+S) jointly contribute 61.3% (R: 37.2%, S: 24.1%), while the enabler panel (E) contributes 38.7%. The R panel exhibits the highest cross-city dispersion in SHAP contributions, reflecting highly differentiated representation of natural endowments within the policy-recognition system. The E panel is relatively homogeneous yet accounts for nearly 40% of total contribution, indicating a systematic supporting role in the learned mapping.

**Feature importance ranking (Panels B1–B2)**. Within the resource base (Panel B1), the top five features (forest coverage rate, elevation range, national sports events, high health-value lakes, surface pressure) jointly contribute 45.8%, with the top ten reaching 64.2%. Forest coverage rate ranks first, accounting for 15.6% of total resource-base attribution. This indicates that forest-related indicators occupy a highly legible position within the current policy-recognition system. The global ranking incorporating enablers (Panel B2) shows urban per capita income (*I*_*k*_≈0.027) and digital economy index (*I*_*k*_≈0.026) leaping to second and third positions behind forest coverage rate, followed by highway length and high-grade scenic area counts. Contrasting B1 and B2 reveals that resource base features concentrate heavily on forest and terrain indicators internally, whereas economic and digitalization variables from enablers enter top positions once incorporated into the global view, pointing to structural differences in how resource-base and enabling variables are represented within the reference-signal mapping.

**Coverage boundary candidate identification (Panel C)**. Two screening criteria are applied at the feature level. A feature is retained as a coverage-boundary candidate if its cross-city CV exceeds the feature-level median (1.22) and its global importance falls within the bottom 50%. Twelve features satisfy both conditions, concentrated in geothermal resources (mineral water zones, hot springs), waterfront and coastal resources (coastlines), traditional Chinese medicine resources (famous practitioners, intangible heritage), and selenium-rich resources. Their common attribute is high spatial agglomeration combined with relatively weak compatibility with standardized, cross-regional evidence requirements. Some cities may possess locally meaningful advantages in these dimensions, but such advantages are less easily represented through cross-regional standardized indicators.

**Contribution concentration (Panel D)**. Individual and cumulative contribution curves for R+S features exhibit a steep initial rise followed by a flattening long tail: the first five features contribute nearly half, with marginal increments declining rapidly thereafter, consistent with Proposition 1 regarding a sparse policy-recognition structure.

Test-period global importance results reveal three structural characteristics of the institutional reference-signal mapping. First, the learned mapping draws on both resource bases and enablers, with neither group negligible in contribution share. Second, resource bases exhibit pronounced internal concentration, with a few highly legible elements contributing disproportionately. Third, certain spatially heterogeneous resources show low global legibility under standardized recognition systems. These results provide empirical baselines for subsequent analysis of non-linear contribution patterns, conditional-attribution patterns, and pathway typology.

### Non-linear contribution patterns and conditional amplification

**Figure 4:**
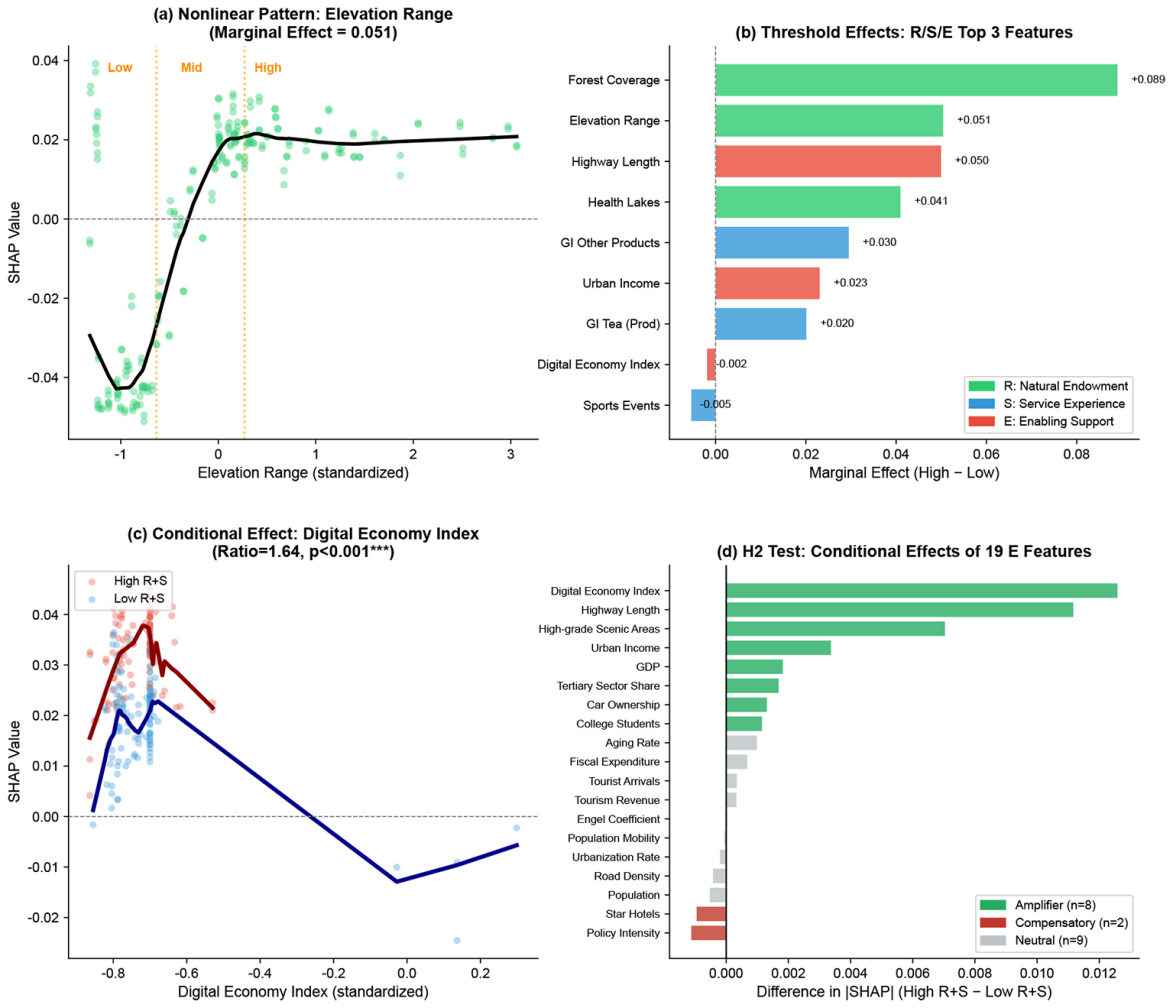
Non-linear SHAP contribution patterns and conditional amplification of enablers. **(a)** SHAP dependence plot for elevation range, illustrating a threshold pattern across low, middle, and high value ranges. **(b)** Marginal effects of representative R/S/E features between high and low value groups, showing threshold-related contribution differences. **(c)** Conditional SHAP pattern of the digital economy index by high versus low R+S resource-base strength, showing stronger positive attribution in the high-resource-base group. **(d)** Conditional effects of 19 enabler features, distinguishing amplifier, compensator, and neutral attribution patterns based on differences between high and low R+S groups.

Figure 4 presents non-linear SHAP contribution patterns of key features and conditional-attribution patterns of enablers. Dependence plots are interpreted as attribution patterns within the learned mapping rather than as causal dose–response relationships.

Figure 4a takes elevation range as an example. The SHAP dependence plot shows a clear threshold pattern. Below the 33rd percentile, contributions are mostly negative. After crossing this threshold, they turn sharply positive and rise steeply. Above the 67th percentile, the marginal contribution flattens. Forest coverage rate and high health-value lakes show similar threshold patterns (Figure 4b). Terrain and ecological resources therefore enter the institutional reference-signal mapping mainly above certain intensity levels. Other features behave differently. Geographical indication products on the service-experience side show relatively stable positive linear differences. Sports events and digital economy index instead show near-zero contributions at both ends of their value range. Their attribution patterns are therefore governed by more complex conditional mechanisms rather than by a simple threshold.

The conditional-attribution pattern of digital economy index provides further explanation (Figure 4c). After dividing the sample into high/low groups by resource base strength (R+S) median, SHAP contributions in the high-resource-base group systematically exceed those in the low group (ratio = 1.64, *p* < 0.001), indicating that positive recognition attribution associated with digitalization is not universal across all cities but becomes stronger when resource bases are already strong.

This conditional-attribution pattern is not unique to digital economy index (Table 2, **Panel A**; Figure 4d). Under the stock specification, 8 of 19 E features show stronger attribution in the high-resource-base group, with standardized attribution differences tapering from large (digital economy index) through medium (highway length) to small (high-grade scenic area counts, urban income, GDP, and four others). Two features show a compensation pattern, with stronger attribution in the low-resource-base group. The remaining 9 show no significant differences. The overall pattern is amplifier-dominated, supporting Proposition 2 in the cumulative reference-signal dimension. In the flow model, serving as an auxiliary specification, digital economy index and high-grade scenic area counts likewise function as significant amplifiers, providing supplementary evidence for the cross-scale robustness of conditional-attribution patterns (Table 2, **Panel B**).

**Table 2:** Conditional SHAP contribution differences of enabler features: Stock and flow specifications.

Panel	Feature	High R+S	Low R+S	Diff	Ratio	*p*	Cohen's *d*	Attribution pattern
A. Stock	Digital Economy Index	0.0323	0.0197	+0.0126	1.64	< 0.001	1.432	Amplifier (L)
A. Stock	Highway Length	0.0231	0.0120	+0.0112	1.93	< 0.001	0.529	Amplifier (M)
A. Stock	High-grade Scenic Areas	0.0197	0.0127	+0.0070	1.56	< 0.001	0.464	Amplifier (M)
A. Stock	Urban Income	0.0285	0.0251	+0.0034	1.13	0.032	0.276	Amplifier (S)
A. Stock	GDP	0.0046	0.0028	+0.0018	1.66	0.004	0.357	Amplifier (S)
A. Stock	Tertiary Sector Share	0.0074	0.0057	+0.0017	1.30	0.016	0.262	Amplifier (S)
A. Stock	Car Ownership	0.0059	0.0046	+0.0013	1.29	< 0.001	0.257	Amplifier (S)
A. Stock	College Students	0.0044	0.0032	+0.0012	1.36	0.002	0.263	Amplifier (S)
A. Stock	Star Hotels	0.0034	0.0043	−0.0010	0.78	< 0.001	−0.212	Compensator
A. Stock	Policy Intensity	0.0020	0.0031	−0.0011	0.64	0.006	−0.271	Compensator
B. Flow	Digital Economy Index	0.1702	0.1347	+0.0355	1.26	< 0.001	0.474	Amplifier (S)
B. Flow	High-grade Scenic Areas	0.1962	0.1572	+0.0390	1.25	< 0.001	0.250	Amplifier (S)
B. Flow	Fiscal Expenditure	0.0199	0.0164	+0.0036	1.22	0.004	0.194	Amplifier (S)
B. Flow	Star Hotels	0.0124	0.0098	+0.0026	1.27	0.002	0.194	Amplifier (S)
B. Flow	Population	0.0110	0.0093	+0.0017	1.18	0.024	0.139	Amplifier (S)
B. Flow	Urban Income	0.1391	0.1600	−0.0209	0.87	0.010	−0.177	Compensator
B. Flow	Population Mobility	0.0712	0.0951	−0.0240	0.75	< 0.001	−0.259	Compensator
B. Flow	Tertiary Sector Share	0.0611	0.0786	−0.0174	0.78	< 0.001	−0.281	Compensator
B. Flow	Aging Rate	0.0432	0.0579	−0.0147	0.75	< 0.001	−0.262	Compensator
B. Flow	Urbanization Rate	0.0333	0.0476	−0.0142	0.70	< 0.001	−0.323	Compensator

High/Low groups are defined by the median standardized sum of R+S resources. SHAP values in the stock model are on the original prediction scale, whereas SHAP values in the flow model are on the log-odds scale. The two panels are comparable in direction and pattern, not in direct magnitude. The labels “Amplifier” and “Compensator” describe model-attribution patterns within the recognition-signal mapping and do not denote causal effects on recognition, development, or health outcomes. Statistical significance is based on 1,000 bootstrap resamples.

Natural endowments therefore show clear threshold patterns, while enabler attribution patterns depend on the resource base. Enablers show stronger positive recognition attribution mainly in cities with stronger and more legible resource bases. This pattern supports Proposition 2.

### Heterogeneous resource pathways and diagnostic positions

**Figure 5:**
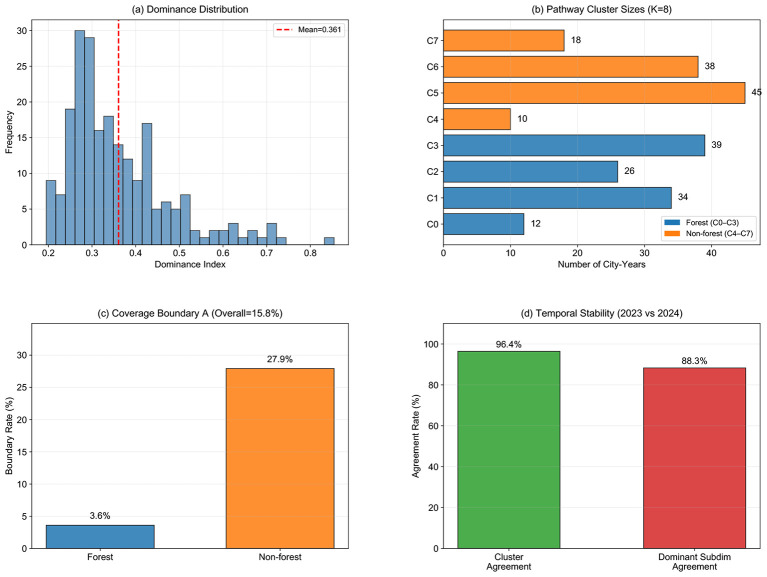
City-level pathway heterogeneity and temporal stability. **(a)** Distribution of the Dominance Index for positive resource-base SHAP contributions; the dashed line indicates the sample mean. **(b)** Sizes of the eight pathway clusters identified by two-stage Ward clustering, distinguishing forest-advantaged clusters from non-forest clusters. **(c)** Coverage-boundary rates for forest and non-forest groups, showing higher boundary exposure among non-forest pathways. **(d)** Temporal stability of pathway classification between 2023 and 2024, reported as cluster-assignment agreement and dominant-subdimension agreement.

Positive reference-signal contribution structures for 222 city-year observations in the test period show clear concentration. The mean Dominance Index is 0.361 and the median is 0.330, far exceeding the uniform benchmark under 11 subdimensions. The mean cumulative contribution share of the top three subdimensions reaches 72.9%, and all observations exceed 50%. These results support Proposition 1 at the city level: positive recognition contributions are typically concentrated around a small number of dominant resource dimensions rather than distributed across balanced multidimensional profiles (see Figure 5).

Single-stage Ward clustering on the full sample reveals that forest-dominated clusters cover a large share of observations, while non-forest configurations are compressed into smaller and less differentiated groups. This indicates that Forest Ecology dominates distances in the contribution space, making it necessary to avoid compressing non-forest heterogeneity into residual groups. To avoid allowing the dominant forest signal to obscure non-forest heterogeneity, pathway typology adopts a two-stage Ward strategy. The sample is first divided into a Forest-advantaged group and a Non-forest group at the median of Forest Ecology positive SHAP contributions, and clustering is then implemented within each group. This procedure yields eight pathways (Table 3).

**Table 3:** Diagnostic profiles of city-level resource pathways based on two-stage Ward clustering.

Cluster name	*N*	Share	Avg Y	Avg Dom	Boundary	Group	Diagnostic position	Typical cities (2024)
C0: Forest Ecology–Cultural Space (+Terrain)	12	5.4%	0.460	0.373	0.0%	Forest	Policy-aligned	Zhangjiajie; Pingxiang
C1: Forest Ecology–Waterscape (+Terrain)	34	15.3%	0.471	0.317	11.8%	Forest	Policy-aligned	Ji'an; Chenzhou; Yuxi
C2: Forest Ecology–Climate (+Terrain)	26	11.7%	0.538	0.301	0.0%	Forest	Policy-aligned	Guangyuan; Lincang; Leshan
C3: Forest Ecology–Climate (+TCM Heritage)	39	17.6%	0.570	0.262	0.0%	Forest	Policy-aligned	Jinhua; Changsha; Wenzhou
C4: TCM Heritage–Food & GI (+Waterscape)	10	4.5%	0.499	0.299	100.0%	Non-forest	Boundary-advantage	Nanjing; Huanggang;
C5: Climate–Cultural Space (+Forest Ecology)	45	20.3%	0.128	0.473	15.6%	Non-forest	Low-readiness	Lianyungang; Bengbu
C6: Food & GI–Cultural Space (+Terrain)	38	17.1%	0.307	0.438	2.6%	Non-forest	Low-readiness	Shaoxing; Dazhou; Wuhu
C7: Waterscape–Terrain (+Cultural Space)	18	8.1%	0.449	0.325	72.2%	Non-forest	Boundary-advantage	Anqing; Hengyang

Avg Y, mean normalized recognition index. Avg Dom, mean share of dominant subdimension contribution. Boundary = share of observations dominated by low-legibility dimensions, defined as the bottom 50% of subdimensions ranked by global importance. Parentheses indicate the third-ranked dimension. Typical cities are the observations nearest to the 2024 cluster centroids. Diagnostic position interprets each cluster relative to the dominant template of the policy-recognition system. Policy-aligned clusters fit highly legible reference channels; low-readiness clusters show limited recognition mainly because of insufficient resource strength or pathway maturity; boundary-advantage clusters possess distinctive but weakly represented dominant dimensions.

The four Forest-advantaged pathways, C0 to C3, are all dominated by Forest Ecology, with secondary differentiation across Cultural Space, Waterscape, Climate, Terrain, and TCM Heritage. Their average institutional reference-index values range from 0.460 to 0.570, and their boundary rates are low, ranging from 0.0% to 11.8%. These pathways are therefore interpreted as policy-aligned positions. Their resource configurations fit the dominant template of the current policy-recognition system, and their leading dimensions are highly legible under standardized evidence requirements.

The four Non-forest pathways, C4 to C7, show more diverse resource combinations and require further diagnostic interpretation. C4 is dominated by TCM Heritage and Food & GI, with Waterscape as the third dimension. Its average institutional reference-index value is relatively high, but its boundary rate reaches 100.0%, indicating that its dominant advantages are locally meaningful yet weakly represented within current recognition templates. C7 is dominated by Waterscape and Terrain, with Cultural Space as the third dimension, and its boundary rate reaches 72.2%. These two clusters are therefore interpreted as boundary-advantage positions. Their difficulty does not lie primarily in the absence of resources, but in the limited institutional legibility of their dominant resource dimensions.

C5 and C6 represent a different diagnostic situation. C5 has the lowest average recognition value and a high dominance index, but its dominant dimensions, Climate and Cultural Space, are not consistently low-legibility dimensions. Its low recognition more likely reflects limited pathway readiness or weak configuration coherence rather than the weak institutional invisibility. C6, dominated by Food & GI and Cultural Space, also shows a relatively low average recognition value but a very low boundary rate of 2.6%. This indicates that its resource dimensions are not outside the current reference template, yet they have not generated strong positive recognition contributions. C5 and C6 are therefore interpreted as low-readiness positions rather than boundary-advantage positions.

Overall, 15.8% of observations fall into coverage-boundary cases. The boundary rate differs sharply between the Forest-advantaged and Non-forest groups: 3.6% in the former and 27.9% in the latter. Within the Non-forest group, boundary observations concentrate mainly in C4 and C7, whose dominant subdimensions include TCM Heritage and Waterscape. This pattern confirms that the institutional coverage boundary is not equivalent to low recognition in general. Some low-recognition cities face pathway-readiness or configuration-coherence constraints, whereas others possess distinctive resource advantages that are weakly translated into recognition signals because their dominant dimensions remain outside the effective coverage of current templates.

Temporal stability tests further support the robustness of the typology. Among the 111 cities appearing in both 2023 and 2024, pathway cluster assignment consistency reaches 96.4%, and dominant subdimension consistency reaches 88.3%. City-based bootstrap testing yields a mean adjusted Rand index of 0.657, with a 95% confidence interval from 0.486 to 0.865. These results indicate moderate-to-high stability under sampling perturbations and suggest that the identified pathways capture relatively stable contribution structures rather than random clustering artifacts.

To make these diagnostic positions more concrete, it is useful to contrast two centroid-near cases. Zhangjiajie, the observation nearest the C0 centroid, illustrates a policy-aligned position: its forest cover, protected mountain landscapes, and administratively classified ecological assets closely match indicators that the current template can measure and compare, including coverage rate, protected-area status, and ecological classification. Its strong recognition therefore reflects the high legibility of the forest-ecology channel.

By contrast, Nanjing, the observation nearest the C4 centroid, illustrates a boundary-advantage position. Its distinctive assets, including traditional Chinese medicine heritage, food- and geographical-indication-based dietary culture, and riparian waterscapes along the Yangtze and Qinhuai systems, are more experiential and service-mediated, making them harder to translate into standardized recognition signals. Although Nanjing is not weakly recognized in absolute terms, with a cluster mean recognition index of 0.499, its dominant advantages fall almost entirely within low-legibility dimensions, and the cluster boundary rate reaches 100.0%. Together, the two cases show that the typology locates cities relative to what the current template can see, rather than ranking them along a single ladder of urban quality.

### Robustness checks

To test whether the diagnostic findings depend on benchmark scaling, forest-theme weighting, or pandemic-period disturbance, three robustness checks are implemented while maintaining strict temporal extrapolation and identical feature preprocessing where applicable (Table 4). For cross-specification comparability, Boundary A in RC1, RC2, and RC3 uses the low-legibility subdimension set defined under the BASE specification. Two auxiliary diagnostics further examine downstream-midstream-upstream heterogeneity and coarse reach-specific annual shocks.

**Table 4:** Robustness checks: test-period performance and structural diagnostics.

Specification	Stock R^2^	Flow PR-AUC	Avg Dom	Avg Top3	Boundary A
BASE (tiered scoring)	0.756	0.503	0.361	72.9%	15.8%
RC1 (count-based)	0.694	0.505	0.362	73.7%	18.9%
RC2 (forest weighted α = 0.5)	0.230	0.253	0.365	74.7%	5.9%
RC3 (exclude 2020-2021)	0.786	0.297	0.380	76.7%	18.0%

Stock R^2^ and Flow PR-AUC are the primary cross-specification comparators. MAE and RMSE are not directly comparable across differently scaled criteria and are therefore reported only in the accompanying text. Avg Dom, mean Dominance Index; Avg Top3, mean cumulative positive SHAP share of the top-three resource-base sub-dimensions. Boundary A is computed using a fixed low-legibility set defined under BASE (tiered scoring), i.e., the bottom 50% of sub-dimensions ranked by BASE global importance Ik, to ensure cross-specification comparability. RC3 excludes 2020–2021 from the training set while keeping the validation year and test period unchanged.

**RC1: criterion scaling replacement**. A count-based specification using cumulative designation counts replaces the tiered scoring criterion to test whether conclusions depend on specific Base-Bonus weighting. The main diagnostic patterns remain largely stable: stock declines modestly (MAE = 0.137, RMSE = 0.166), while the flow specification remains virtually unchanged, with ROC-AUC changing only from 0.746 to 0.747 and PR-AUC from 0.503 to 0.505, contribution concentration and coverage-boundary identification exhibit no directional reversal. Re-testing Proposition 2 conditional-attribution patterns confirms that digital economy index and high-grade scenic area counts retain stronger attribution in the high-resource-base group (p < 0.05), with directions consistent with the BASE specification.

**RC2: forest theme downweighting**. All forest-theme designation weights are multiplied by α = 0.5, and the models are retrained using the same temporal partition and preprocessing procedure. Under the forest-downweighted criterion, stock explanatory power declines substantially, with Stock R^2^ falling to 0.230, and flow discriminatory power also weakens. Yet contribution concentration does not decrease, indicating that sparse recognition patterns are not solely an artifact of forest-theme weighting but remain stable within the learned reference-signal contribution structure. Conditional-attribution testing likewise confirms that digital economy index and high-grade scenic area counts maintain stable high-resource-base directions (*p* < 0.05). Boundary A, however, falls from 15.8% to 5.9%. Weakened forest-theme signals reduce model discriminability along the high-legibility and low-legibility gradient, making coverage-boundary triggering less sufficient. RC2 therefore resembles a scenario in which compressed benchmark information content makes the reference-signal mapping harder to learn, rather than a scenario in which contribution structures undergo directional reversal.

**RC3: pandemic-period sensitivity**. The model is retrained after excluding the 2020–2021 outbreak years from the training set, while the validation and test periods remain unchanged. The BASE rerun reproduces the original diagnostics, confirming that the same preprocessing and diagnostic pipeline is used. After excluding the outbreak years, stock prediction remains stable (R^2^ = 0.786, MAE = 0.074, RMSE = 0.091). The flow specification becomes weaker (PR-AUC = 0.297), but the main structural diagnostics do not reverse. Positive contributions remain concentrated, with a mean dominance of 0.380 and a mean top-three share of 76.7%. Forest coverage retains a central position within resource-base attribution at 16.3%. Key enabling variables keep the same high-vs.-low R+S attribution direction. Digital economy index (ratio = 1.30), highway length (ratio = 2.22), and high-grade scenic areas (ratio = 1.63) all show stronger mean absolute SHAP attribution in the high-resource-base group. The overall Boundary A rate is 18.0% (Table 4). When decomposed by pathway type, boundary observations remain more concentrated in non-forest pathways (26.1%) than in forest pathways (9.9%).

As a regional directional check, the 222 test-period city-year observations are further stratified into downstream, midstream, and upstream subsamples to address the possibility that pandemic-period shocks affected cities differently across the Yangtze River Economic Belt. This check uses the saved test-period SHAP attributions and pathway assignments rather than region-specific retraining, and only sign- and rank-level diagnostics are reported because each regional subsample is limited in size. Its purpose is to examine whether the main diagnostic directions reverse under regional heterogeneity, not to compare predictive accuracy. Forest Ecology remains highly visible across the three reaches. It ranks first in the downstream and midstream subsamples and second in the upstream subsample, with resource-base shares of 22.8%, 20.3%, and 20.0%. Because these shares remain close across reaches, the second-place ranking upstream reflects locally stronger terrain and climate visibility rather than a departure from the forest-centered structure. Boundary observations also remain concentrated in non-forest pathways across all three reaches. No boundary cases appear within forest pathways in the downstream or upstream subsamples, while the non-forest boundary rates are 27.4% and 9.1%. In the midstream subsample, the non-forest boundary rate is also higher than the forest-pathway rate, at 44.4% vs. 8.5%. Digital economy index and high-grade scenic-area counts keep their high-vs.-low R+S attribution direction across the three reaches. Highway length varies by reach and shows a weaker contribution pattern in the downstream subsample. Transport infrastructure may therefore operate differently where baseline accessibility is already high.

As a further auxiliary check, a reduced-form OLS stock model is estimated on the full city-year panel using the same 58 standardized explanatory variables together with downstream-, midstream-, and upstream-by-year fixed effects. This specification absorbs coarse reach-specific annual shocks, although it does not replace direct city-level pandemic-severity controls. Forest coverage remains positive and significant (coef. = 0.067, *p* = 0.011), ranking third among the 39 R+S variables by absolute standardized coefficient. This result suggests that the forest-centered visibility pattern is not eliminated after absorbing coarse reach-specific annual shocks. High-vs.-low R+S coefficient-weighted contrasts in this auxiliary linear specification are also directionally consistent for digital economy index, highway length, and high-grade scenic areas. The non-linear SHAP-based BASE model remains the primary evidence for conditional amplification.

Across all checks, the sparse contribution structure and the main diagnostic patterns do not reverse. This holds under alternative scoring scales, forest-theme downweighting, pandemic-period sensitivity, upstream-midstream-downstream stratification, and coarse reach-specific annual shock absorption. RC3 is interpreted as a pandemic-period sensitivity check rather than as a replacement for the BASE specification. After excluding the outbreak years, stock prediction remains stable, while flow discrimination weakens. This weakening means that marginal-entry prediction should be interpreted with caution under the reduced training window. The main structural diagnostics nevertheless remain stable. Positive contributions stay concentrated, forest coverage retains a central position within resource-base attribution, and boundary observations remain more concentrated in non-forest pathways than in forest pathways. The auxiliary regional and reach-by-year checks further indicate that these core patterns are not removed by regional heterogeneity or coarse regional-time shocks, although highway length varies by reach.

## Discussion

### Recognition is uneven across health-promoting resource configurations

This study examines how heterogeneous city-level health-promoting resource configurations are represented within a selective but observable policy-recognition system. The empirical results show that policy-recognition records should not be read as neutral measures of comprehensive health-promoting value. Rather, they provide policy-recognition signals that reflect how current standardized recognition templates make some resource configurations more legible than others. This distinction is important for public health because health-promoting environments are formed not only by forests and ecological spaces, but also by waterscapes, terrain, climate, geothermal environments, traditional health practices, dietary resources, cultural-spiritual spaces, sports and leisure resources, service capacity, and access conditions. These resources may support restoration, physical activity, preventive care, social interaction, dietary practice, and mental wellbeing, but they are not equally easy to document, audit, classify, and compare within standardized recognition systems.

Recognition is first characterized by a sparse contribution structure. Positive recognition contributions are concentrated around a limited set of highly legible indicators, especially forest-related indicators. Forest coverage rate alone accounts for 15.6% of total resource-base attribution, indicating that forest resources occupy a highly legible position within the current policy-recognition system. Evidence from China also suggests that national forest-city initiatives can improve residents' health through environmental and behavioral pathways (25). Forest indicators are especially compatible with standardized evidence requirements because they can be measured through coverage rate, spatial boundaries, ecological classification, and administrative records, which strengthens their legibility within the recognition system without implying intrinsic superiority over other health-promoting resources (16–18). Rather than serving only as a robustness check, the RC2 result reveals a substantive feature of the recognition system: the benchmark itself is strongly forest-loaded. This structure is not a modeling artifact but the sparse, forest-centered legibility that this study seeks to diagnose.

Enablers show a conditional rather than universal role. Transportation, digitalization, economic foundations, service capacity, and policy supply do not contribute uniformly across all cities within the reference-signal mapping. Their positive recognition contributions become stronger mainly when cities already possess relatively strong and legible resource bases. Enablers therefore work as conditional amplifiers rather than universal substitutes for weak resource conditions. This pattern is consistent with the complementary-assets logic in which resources generate stronger value when combined with appropriate supporting capabilities (15, 26). The amplification pattern refers to stronger representation within the reference-signal mapping, not to causal effects on health outcomes.

Pathway analysis further reveals multiple resource configurations under unequal institutional legibility. The eight identified pathways do not form a simple hierarchy of urban quality or health-promoting value. Instead, they indicate different diagnostic positions relative to the current policy-recognition system. Forest-anchored clusters occupy policy-aligned positions because their dominant dimensions fit highly legible evidence templates. C5 and C6 are interpreted as low-readiness positions because their relatively weak recognition appears to reflect insufficient resource depth, configuration coherence, or pathway maturity rather than weak institutional legibility. C4 and C7 are interpreted as boundary-advantage positions because their dominant dimensions, including TCM Heritage and Waterscape, are locally meaningful but weakly represented within current standardized templates.

This distinction is central to the contribution of the study. Weak recognition should not be mechanically interpreted as low health-promoting value. Some cities may indeed face resource-depth or pathway-readiness constraints. Others may possess distinctive health-promoting assets that remain weakly represented because their dominant advantages are difficult to translate into standardized evidence. The contrast between low-readiness pathways and boundary-advantage pathways shows why a single recognition-oriented interpretation would be misleading. The analytical task is not to identify which cities should pursue recognition more aggressively, but to diagnose whether weak representation reflects resource constraints, pathway immaturity, or limited template sensitivity.

These findings extend configurational thinking by showing that health-promoting resource development cannot be reduced to a single dominant template. Different configurations may support different forms of restoration, prevention, activity, service experience, and wellbeing. However, the policy-recognition system does not read all configurations with equal sensitivity. Forest-based pathways are strongly represented because they fit current evidence templates, whereas other pathways, such as those based on therapeutic waterscapes, geothermal resources, traditional health practices, dietary cultures, or cultural-spiritual resources, may remain close to the institutional coverage boundary. This creates a potential gap between the distribution of locally meaningful health-promoting assets and the distribution of policy recognition.

From an environmental health perspective, the findings suggest that standardized recognition systems may unintentionally privilege certain types of health-promoting environments while under-representing others. Green-space and forest-based resources are highly legible, whereas blue-space, geothermal, dietary, traditional-service, and experiential resources may be harder to standardize. This does not mean that standardized recognition systems are inappropriate, since such systems necessarily require comparability and accountability. However, it does show that recognition records should be interpreted as selective policy-recognition signals rather than comprehensive evaluations of place-based health-promoting potential.

### Theoretical implications

#### Reframing recognition records as policy-recognition signals

Reframing recognition records is the first theoretical move. In many studies, designation or recognition records are implicitly treated as quality labels, performance proxies, or indicators of development success. This study argues that recognition records are better understood as policy-recognition signals. They are observable, comparable, and analytically useful, but they are also selective. Their value lies not in measuring true health-promoting value, but in revealing how current recognition templates represent heterogeneous resource configurations. This reframing separates resource value from recognized value. A city may possess meaningful health-promoting resources but remain weakly recognized if those resources cannot be documented, audited, or compared through existing templates.

Conversely, strong recognition may reflect template fit rather than superiority across all dimensions of public health value. Recognition records therefore should not be interpreted as direct measures of population-health benefit, comprehensive resource value, or policy success. They are institutional signals that reveal the selective logic of standardized recognition.

This perspective also clarifies how institutional legibility relates to legitimacy. Institutional theory has long argued that organizations and places seek legitimacy by conforming to institutional expectations (16, 18). This study shifts attention to a prior condition. Before legitimacy can be granted, local advantages must first become legible. The institutional coverage boundary marks the point at which locally meaningful advantages fail to enter the recognition field, because the evidentiary template cannot read them. Weak legibility is therefore not the same as lack of legitimacy. It can occur before formal legitimacy evaluation begins.

#### From resource inventories to contribution diagnosis

The results also shift resource-based analysis from inventory description to contribution diagnosis. Traditional resource-based analysis often begins by asking what resources a place possesses. However, resource possession alone does not explain how different cities are represented within standardized recognition systems. This study moves from resource inventories to contribution diagnosis by asking which resources become positive reference-signal contributors under current templates, which resources remain weakly represented, and how these patterns differ across cities.

The R-S-E framework supports this shift by distinguishing natural endowment resources, service-experience resources, and enablers according to their functional roles. Natural endowments provide ecological and spatial foundations for health promotion. Service-experience resources translate environmental and cultural assets into usable health-promoting experiences. Enablers support access, implementation, and service transformation. The empirical results show that these categories do not contribute in a simple additive manner. Instead, their contributions are conditional, non-linear, and unevenly legible within the policy-recognition system.

The combination of XGBoost and SHAP provides a methodological basis for this contribution diagnosis. XGBoost captures non-linear and interactive patterns between resource configurations and the policy-recognition system, while SHAP decomposes the learned mapping into feature-level and city-level contribution structures (23, 24). The analytical value of this approach lies not in prediction for its own sake, but in making the policy-recognition structure traceable. It becomes possible to identify sparse recognition patterns, conditional amplification, pathway heterogeneity, and potential coverage-boundary cases.

The results refine the complementary-assets logic by extending it from resource deployment to institutional representation. Enablers show stronger representation alongside resources that can already be read by the recognition system, but they cannot fully compensate for resource bases that remain weakly legible under current templates.

### Operationalizing the institutional coverage boundaries

The institutional coverage boundary is also operationalized as a measurable diagnostic construct. Existing institutional research has emphasized that evaluation systems, certification schemes, and recognition programs are selective (17), but it is often difficult to identify where the boundary of recognition actually lies. This study transforms the institutional coverage boundary from a conceptual observation into a measurable diagnostic construct.

Operationally, the boundary is identified through the mismatch between local dominance and global legibility. If a city's dominant positive contribution dimension belongs to a globally low-legibility set, the city is identified as a potential coverage-boundary case. This does not mean that the resource is unimportant. It means that the city's dominant advantage is weakly represented within the current policy-recognition mapping. By aggregating these cases into pathways, the study further distinguishes policy-aligned, low-readiness, and boundary-advantage positions.

This distinction advances the interpretation of weak recognition. Low representation may result from at least two different sources. One is insufficient resource depth or pathway maturity, as reflected in low-readiness pathways. The other is weak institutional legibility, as reflected in boundary-advantage pathways. The framework therefore distinguishes resource constraints from institutional under-recognition, two sources of weak representation that would otherwise be confounded.

The concept has broader relevance beyond the empirical setting of China's wellness-related recognition programs. Standardized assessment and recognition systems are widely used in environmental health policy, healthy-city programs, green and blue space planning, and land use planning that integrates health-promoting environmental assets into spatial governance. In all these fields, evaluation templates must balance comparability with sensitivity to local heterogeneity. The institutional coverage boundary provides a way to examine where this balance becomes uneven and where certain locally meaningful resource configurations become systematically under-represented.

### Environmental health policy implications

For local policy use, the three diagnostic positions should be read as interpretive judgments rather than as a ranking of cities. The first question is whether a city's main advantages are already legible to existing recognition templates, such as forest ecology, terrain, climate, or administratively classified ecological assets. Cities in this policy-aligned position can work through existing recognition channels while continuing to strengthen accessibility, environmental monitoring, service quality, and public-health relevance.

A second question is whether the city also holds distinctive assets that are harder to document, such as traditional medicine services, therapeutic waterscapes, geothermal resources, food-based wellness, or culturally embedded wellness resources. These assets should not be crowded out simply because another resource type fits the current template more easily. Where such low-legibility resources are locally strong and form the city's main advantage, the city is better understood as a boundary-advantage case. The policy task is not to imitate forest-centered templates, but to make these assets more credible through service access, safety standards, professional validation, and user feedback.

The low-readiness position requires a different response. A city may hold resources that current templates can in principle recognize, yet still fail to generate strong recognition because those resources are thin, immature, or not organized into a coherent service pathway. For such cities, the main constraint is not template coverage but resource depth and pathway maturity. Resource consolidation, service organization, and quality control should therefore come before any push for stronger recognition.

For recognition-system designers, the findings support modular and multi-track qualification pathways. A feasible design would retain a common baseline review covering safety management, inclusive accessibility, environmental quality monitoring, service capacity, and public-health relevance, while allowing cities to enter through parallel resource tracks such as forest ecology, blue-space restoration, geothermal environments, traditional health services, food-based wellness, and sports-and-leisure spaces. If policy goals include diversified, equitable, and resilient health-promoting development, such a design would preserve cross-track comparability while reducing the chance that only the easiest-to-audit resource types become visible (12, 20). This concern is especially relevant for environmental health policy, because health-promoting environments include not only green spaces but also blue spaces, therapeutic environments, traditional service systems, and social spaces that are meaningful yet harder to standardize. For example, a city with strong therapeutic waterscapes and traditional health services but limited forest cover could be assessed through the common baseline plus blue-space and traditional-health tracks, rather than against a forest-centered template.

The coverage-boundary diagnosis also has a measurement implication. Many under-read resources are currently captured mainly through counts or presence indicators, including geothermal hot springs, mineral-water and selenium-enriched zones, TCM heritage, and geographical-indication products. These measures show that a resource exists, but they say less about whether it has become an accessible, credible, and health-promoting service. Future certification systems could therefore supplement resource inventories with indicators of access, service quality, safety, and influence. For example, traditional medicine resources could be assessed through the accessibility of graded TCM wellness service points, geothermal resources through certified therapeutic-service density, food-based resources through traceability coverage and market influence, and waterscape resources through safe recreational accessibility, so that service-based and experiential resources can enter the recognition system in a more comparable form.

The public-health value of this diagnostic framework lies in separating template fit from health-promoting potential. A city with weak recognition may need resource consolidation, service-pathway formation, or a more suitable recognition track. These are different problems that call for different responses. Decisions about whether recognition templates should be broadened, and how they should balance comparability with local specificity, require deliberation among policymakers, public-health professionals, service providers, local communities, and users.

### Limitations and future research

This study has several limitations. First, it examines policy-recognition signals rather than realized public-health outcomes. The benchmark used in supervised learning is not a direct measure of health improvement, service quality, market performance, or comprehensive resource value, and SHAP decomposition reflects model attribution rather than causal effects. This design is partly constrained by data availability: no unified cross-city, city-year measure of realized wellness value is currently available at the prefecture-city scale. The internally constructed recognition benchmark is therefore a necessary design under this data constraint rather than a chosen substitute for an available external criterion. Future research could link recognition structures with external health indicators to examine whether institutional visibility aligns with realized public-health benefits.

Second, the empirical conclusions are situated in the Yangtze River Economic Belt. The Belt contains substantial heterogeneity across eastern, central, and western China, which makes it suitable for diagnosing recognition asymmetries within a diverse regional system. However, it does not represent all of China. Other regions may differ in resource endowments, policy priorities, administrative routines, and market maturity, so the generalizability of the forest-centered visibility structure and coverage-boundary patterns requires further comparative research.

Third, the temporal window has limits. The test period contains only two years, 2023 and 2024, with 222 city-year observations, and the study period also covers the COVID-19 outbreak and recovery years. Strict temporal extrapolation reduces information leakage, and robustness checks suggest that the main structural diagnostics are not eliminated by excluding 2020–2021 from training, by coarse upstream-midstream-downstream heterogeneity, or by regional-time shocks. Even so, the models do not include direct city-level pandemic-severity measures, such as confirmed cases or lockdown duration. Longer panels and more detailed pandemic-severity data would allow stronger tests of temporal persistence and city-specific pandemic disturbance.

Finally, the unit of analysis is the prefecture-level city. This scale matches the administrative level at which many recognition and policy processes operate, but it masks within-city spatial heterogeneity. Finer-scale research could examine whether institutional legibility and coverage-boundary patterns appear differently across districts, counties, communities, scenic areas, and ecological zones.

## Conclusions

This study examined how standardized recognition systems represent heterogeneous city-level health-promoting resource configurations. Using panel data from 111 cities in the Yangtze River Economic Belt from 2017 to 2024, it constructed a policy-recognition benchmark from national and provincial designation records and applied XGBoost with SHAP decomposition under strict temporal extrapolation. The analysis focuses on institutional visibility within recognition systems, tracing how current templates make some health-promoting resource configurations easier to recognize than others.

The results show a sparse recognition structure. Positive recognition contributions are concentrated around a limited set of highly legible indicators, with forest coverage occupying a particularly prominent position. This prominence does not mean that forest-based resources are intrinsically superior to other health-promoting resources. It reflects their strong compatibility with standardized evidence templates. Forest-related resources are easier to measure, audit, classify, and compare across cities, which allows them to generate stronger positive contributions within the current recognition system.

The results also show that enablers play conditional roles. Transportation, digitalization, economic foundations, service capacity, and policy supply show stronger recognition attribution mainly when cities already have relatively strong and legible resource bases. Where resource bases are weak or difficult to read, enablers make more limited contributions or work only as partial compensators. Their value is therefore closely tied to resource depth, pathway coherence, and institutional legibility.

The pathway analysis identifies eight city-level clusters and interprets them through three diagnostic positions. Forest-anchored clusters are generally policy-aligned because their dominant dimensions fit the current recognition template. C5 and C6 are interpreted as low-readiness positions because their weak representation is associated more with limited pathway maturity, configuration coherence, or resource depth than with weak institutional legibility. C4 and C7 are interpreted as boundary-advantage positions because their dominant dimensions, including TCM Heritage and Waterscape, fall into low-legibility sets despite meaningful local advantages.

The institutional coverage boundary helps distinguish different sources of weak representation. Some cities remain weakly represented because pathway readiness or service capacity is insufficient. Others possess distinctive health-promoting resources but remain weakly represented because their dominant advantages are difficult to translate into standardized evidence. This distinction matters for both city strategy and policy interpretation. Weak representation should not be equated with low value, and it should not automatically push cities toward dominant templates.

The study contributes a diagnostic approach for examining recognition asymmetry in environmental health policy and the governance of health-promoting environments. By treating designation records as policy-recognition signals and operationalizing the institutional coverage boundary, it shows how explainable machine learning can trace uneven institutional legibility across diverse health-promoting resource configurations. Future research can extend this approach by linking recognition structures with external health outcomes, examining finer spatial scales, and comparing recognition systems across public-health and environmental-governance contexts.

## Data Availability

The raw data supporting the conclusions of this article will be made available by the authors, without undue reservation.
