# Supplementary material for: Diagnostic analysis of wellness tourism certification bias and public health resource mismatch
Source: Front Public Health. 2026 Jul 14;14:1870963. doi: 10.3389/fpubh.2026.1870963 (PMC13408250; doi:10.3389/fpubh.2026.1870963)
Supplement: Supplementary file 1 [file Supplementary_File_1.docx]

**Appendix**

**Appendix A1: Construction and Validation of Policy Supply Intensity**

The keyword dictionary is organized into four layers. The first layer anchors the core health-and-wellness meaning of the dictionary, and the remaining three layers correspond to the natural-endowment (R), service-experience (S), and enabling-policy (E) dimensions of the analytical framework. Only annual government work reports were used as the corpus, because the release, disclosure, and archiving of specialized policy documents vary across cities, and a single corpus maintains comparability across city-year observations. The full dictionary and its matching rules are listed below.

| **Layer** | **Terms** | **Matching rule** |
| --- | --- | --- |
| Core health-and-wellness anchor | 康养; 康养旅游; 康养产业; 康养基地; 康养小镇; 康养示范; 健康旅游; 健康产业; 健康促进; 健康城市; 健康中国; 医养结合; 医养康养; 养生保健; 康复疗养; 休闲度假; 度假旅游 | Count complete multi-character terms. These terms anchor the health-and-wellness meaning of the dictionary. |
| Natural-endowment discourse (R) | 森林康养; 森林旅游; 生态康养; 生态旅游; 湿地; 温泉; 地热; 避暑旅游; 气候康养; 富氧; 负氧离子; 自然保护地; 国家公园; 风景名胜区; 绿道; 公园城市 | Count direct compound terms. Generic ecological words are counted only when the surrounding expression refers to wellness, tourism, restoration, or health-promotion resources. |
| Service-experience discourse (S) | 中医药健康旅游; 中医药; 中医; 传统医学; 医养结合; 康复; 康复疗养; 温泉疗养; 运动休闲; 体育旅游; 全民健身; 健身休闲; 食养; 药膳; 富硒; 绿色食品; 地理标志; 文化体验 | Count direct compound terms or clearly health-and-wellness-related contexts. Generic medical, sports, cultural, or food terms are excluded when they refer only to general administration. |
| Enabling and policy-support discourse (E) | 康养产业发展; 文旅融合; 农旅融合; 旅游度假区; 示范创建; 品牌建设; 产业扶持; 政策扶持; 专项资金; 招商引资; 基础设施建设; 交通便利; 智慧旅游; 数字赋能; 养老服务; 养老产业 | Count enabling terms only when they relate to health-and-wellness, tourism, elderly care, health promotion, or related destination-development contexts. Generic infrastructure or investment is excluded. |

Note. Dictionary terms are listed in their original Chinese form to preserve matching reproducibility against the Chinese-language corpus, since matching is performed directly on the Chinese reports through Chinese word segmentation. English layer names and matching rules are provided for interpretability. The letters R, S, and E denote the natural-endowment, service-experience, and enabling-policy dimensions of the analytical framework.

Multi-character terms were added to the Chinese word-segmentation dictionary before matching. Policy-attention density was calculated as dictionary hits divided by the valid segmented word count of each report and multiplied by 1,000, with punctuation, whitespace, and pure numerical tokens excluded from the denominator. The raw density was transformed using log(1+x) and standardized with training-period parameters before model estimation.

To validate matching precision, a targeted random sample of 100 city-year reports, covering 20 cities across 5 years, was inspected. Matched terms were manually checked to determine whether they referred to health-and-wellness policy attention rather than general urban governance, social security, environmental protection, medical pricing, or infrastructure discourse. The inspection results were tabulated in Python, and the matched-term accuracy exceeded 90 percent.

**Appendix A2: Variable Definitions, Descriptive Statistics, and Coefficients of Variation**

| Block | Sub-dimension | Variable (Definition and Unit) | Mean | Std. Dev | Min | Max | CV |
| --- | --- | --- | --- | --- | --- | --- | --- |
| R: Natural Endowment | **R1: Geothermal & Hot Spring** | Geothermal Hot Springs (count) | 11.10 | 21.11 | 0.00 | 169.00 | 1.90 |
|  |  | Mineral Water Zone (Binary: 1=Yes) | 0.05 | 0.21 | 0.00 | 1.00 | 4.61 |
|  | **R2: Forest & Ecological** | Forest Coverage Rate (%) | 46.54 | 17.86 | 9.57 | 82.90 | 0.38 |
|  |  | Annual Mean NDVI (Index) | 0.59 | 0.08 | 0.33 | 0.77 | 0.14 |
|  |  | Nature Reserves (count) | 7.30 | 10.41 | 0.00 | 69.00 | 1.43 |
|  |  | National Wetland & Geological Parks (count) | 2.36 | 2.94 | 0.00 | 29.00 | 1.25 |
|  |  | Biological Species Richness (count) | 7441 | 19022 | 17 | 205860 | 2.56 |
|  | **R3: Topographic** | Mean Elevation (m) | 469.35 | 581.03 | 7.34 | 2584.79 | 1.24 |
|  |  | Elevation Range (m) | 1575.64 | 1190.18 | 8.82 | 5217.00 | 0.76 |
|  |  | Mean Slope (degree) | 11.37 | 6.36 | 1.60 | 27.14 | 0.56 |
|  | **R4: Climate** | Comfortable Temperature Days (days/year) | 98.26 | 28.63 | 37.00 | 251.00 | 0.29 |
|  |  | Precipitation-free Days (days/year) | 220.15 | 27.36 | 118.00 | 299.00 | 0.12 |
|  |  | Annual Sunshine Duration (hours) | 2565.54 | 306.14 | 1834.03 | 3676.81 | 0.12 |
|  |  | Total Solar Shortwave Radiation (MJ/m²) | 5045.22 | 509.12 | 4071.64 | 6964.08 | 0.10 |
|  |  | Mean Relative Humidity (%) | 75.15 | 3.76 | 54.19 | 84.69 | 0.05 |
|  |  | Mean Surface Atmospheric Pressure (hPa) | 981.47 | 56.57 | 766.67 | 1017.17 | 0.06 |
|  | **R5: Water Landscape** | High Wellness-Value Lakes (count) | 23.24 | 16.67 | 2.00 | 126.00 | 0.72 |
|  |  | River Network Density (km/km²) | 0.43 | 0.92 | 0.07 | 6.00 | 2.16 |
|  |  | Coastline Length (km) | 74.50 | 309.41 | 0.00 | 2447.00 | 4.15 |
|  | **R6: Env. Quality & Special** | Air Quality Index (AQI) | 68.77 | 13.11 | 36.22 | 111.65 | 0.19 |
|  |  | Selenium-enriched Zone (Binary: 1=Yes) | 0.14 | 0.35 | 0.00 | 1.00 | 2.44 |
| S: Service & Experience | **S1: Medical & Rehabilitation** | Hospital Bed Capacity (count) | 33578 | 29602 | 4452 | 231900 | 0.88 |
|  |  | Licensed Physicians (count) | 15243 | 15237 | 2067 | 108700 | 1.00 |
|  | **S2: Traditional Medicine** | National TCM Masters (count) | 0.68 | 2.10 | 0.00 | 12.00 | 3.09 |
|  |  | Intangible Heritage Items - Medicine (count) | 2.81 | 4.32 | 0.00 | 39.00 | 1.54 |
|  |  | Agri-GI Products - Medicinal Herbs (count) | 0.41 | 0.96 | 0.00 | 6.00 | 2.36 |
|  |  | National GI Products - Medicinal Herbs (count) | 0.84 | 1.56 | 0.00 | 9.00 | 1.85 |
|  | **S3: Spiritual & Cultural** | Confucian Cultural Sites (count) | 36.95 | 38.24 | 0.00 | 210.00 | 1.04 |
|  |  | Buddhist Temples (count) | 159.54 | 210.82 | 0.00 | 1435.00 | 1.32 |
|  |  | Taoist Temples (count) | 43.03 | 112.83 | 0.00 | 1026.00 | 2.62 |
|  |  | Red Revolutionary Cultural Sites (count) | 1.68 | 2.05 | 0.00 | 14.00 | 1.22 |
|  |  | Historic & Cultural Towns/Villages (count) | 2.66 | 3.71 | 0.00 | 21.00 | 1.40 |
|  |  | Traditional Villages (count) | 28.41 | 46.62 | 0.00 | 309.00 | 1.64 |
|  |  | Intangible Heritage Items - Non-Medicine (count) | 29.26 | 26.78 | 3.00 | 245.00 | 0.92 |
|  | **S4: Food & Nutrition** | Agri-GI Products - Tea (count) | 0.99 | 1.72 | 0.00 | 11.00 | 1.75 |
|  |  | National GI Products - Tea (count) | 0.81 | 1.30 | 0.00 | 8.00 | 1.60 |
|  |  | Agri-GI Products - Other (count) | 5.99 | 6.96 | 0.00 | 62.00 | 1.16 |
|  | **S5: Sports & Leisure** | National-level Sports Events (events/year) | 2.26 | 1.93 | 0.00 | 16.00 | 0.85 |
|  |  | Urban Built-up Green Coverage Rate (%) | 41.99 | 3.47 | 27.20 | 64.78 | 0.08 |
| E: Enabler | **E1: Economic Foundation** | Gross Domestic Product (100 million CNY) | 43.76 | 58.77 | 3.39 | 539.27 | 1.34 |
|  |  | Tertiary Industry Share of GDP (%) | 48.65 | 7.33 | 30.21 | 78.20 | 0.15 |
|  |  | Local Fiscal General Budget Expenditure (100 million CNY) | 6.90 | 9.79 | 1.16 | 98.75 | 1.42 |
|  |  | Urban Per Capita Disposable Income (10,000 CNY) | 4.37 | 1.26 | 2.29 | 9.31 | 0.29 |
|  |  | Urban Resident Engel Coefficient (Ratio) | 0.33 | 0.05 | 0.24 | 0.57 | 0.16 |
|  |  | Digital Economy Development Index (0-1) | 0.04 | 0.06 | 0.00 | 0.41 | 1.51 |
|  | **E2: Social Foundation** | Regular Higher Education Enrollment (count) | 135863 | 228179 | 4760 | 1443000 | 1.68 |
|  |  | Permanent Resident Population (million) | 5.09 | 4.27 | 1.06 | 32.13 | 0.84 |
|  |  | Population Mobility Rate (%) | -3.37 | 20.10 | -32.30 | 71.28 | / |
|  |  | Aging Rate (Population Aged 65+, %) | 14.84 | 3.12 | 8.58 | 23.40 | 0.21 |
|  |  | Urbanization Rate (%) | 60.34 | 12.01 | 33.00 | 90.00 | 0.20 |
|  | **E3: Transport Infrastructure** | Road Network Density (km/km²) | 1.47 | 0.45 | 0.44 | 2.89 | 0.30 |
|  |  | Highway Mileage (km) | 513.37 | 398.70 | 16.50 | 4142.00 | 0.78 |
|  |  | Private Car Ownership (10,000 vehicles) | 108.02 | 124.23 | 6.07 | 955.99 | 1.15 |
|  | **E4: Tourism Capacity** | Tourism Revenue (100 million CNY) | 782.97 | 831.49 | 29.00 | 5800.00 | 1.06 |
|  |  | Tourist Arrivals (10,000 visitors) | 7152 | 6669 | 490 | 65700 | 0.93 |
|  |  | Star-rated Hotels (count) | 31.48 | 29.29 | 2.00 | 229.00 | 0.93 |
|  |  | High-grade Scenic Areas (4A+5A) (count) | 15.92 | 15.84 | 0.00 | 176.00 | 1.00 |
|  | **E5: Policy Supply** | Wellness Tourism Policy Supply Intensity (‰) | 2.03 | 0.82 | 0.25 | 7.12 | 0.41 |

Note. This table presents the definitions, measurement units, and descriptive statistics for all 58 feature variables used in the study. The Coefficient of Variation (CV) indicates the degree of spatial heterogeneity across the 111 cities. $N=888$ (111 cities × 8 years); CV for Population Mobility Rate is not reported due to the negative mean value. All statistics are calculated based on the raw values before standardization.
